# Supplementary material for: Deep sequencing reveals a DAP1 regulatory haplotype that potentiates autoimmunity in systemic lupus erythematosus
Source: Genome Biol. 2020 Nov 19;21:281. doi: 10.1186/s13059-020-02184-z (PMC7677828; doi:10.1186/s13059-020-02184-z)

Fig. S1. DAP1 genetic association with SLE

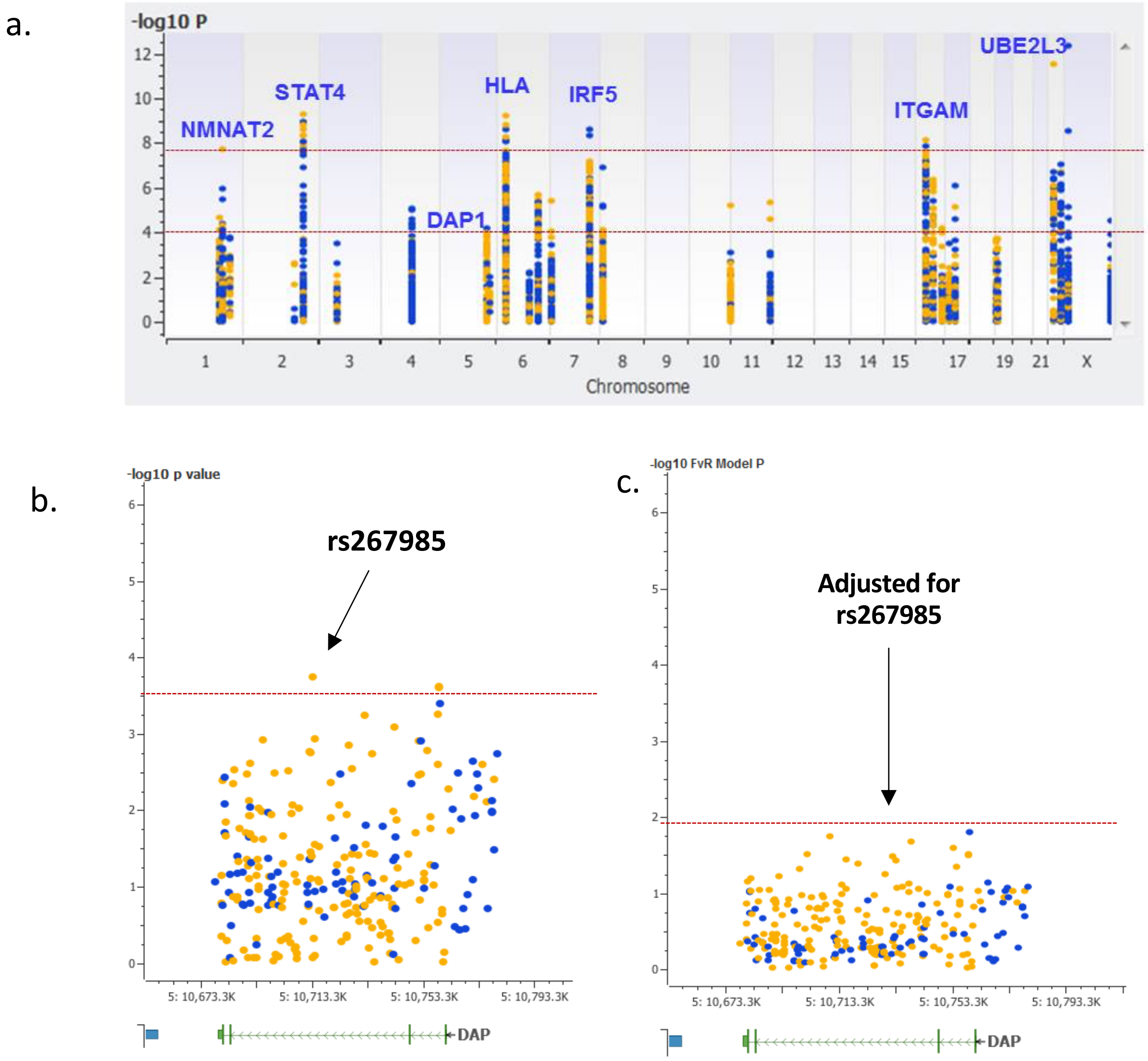

Fig. S1. Manhattan plot showing association of DAP1 gene polymorphisms with SLE. Panel a show a Manhattan plot on SLE association. X axis show chromosome number and y-axis show –log10p value of SLE association. Panel b and c shows association status before and after conditioning on peak SNP rs267985, respectively. Each dot represent an individual SNP, with yellow color indicating a potential functional variant based on ENCODE and RegulomeDB databases, and blue color represent unannotated variants.

Fig. S2. Genomic position of 19 top SLE associated DAP1 variants

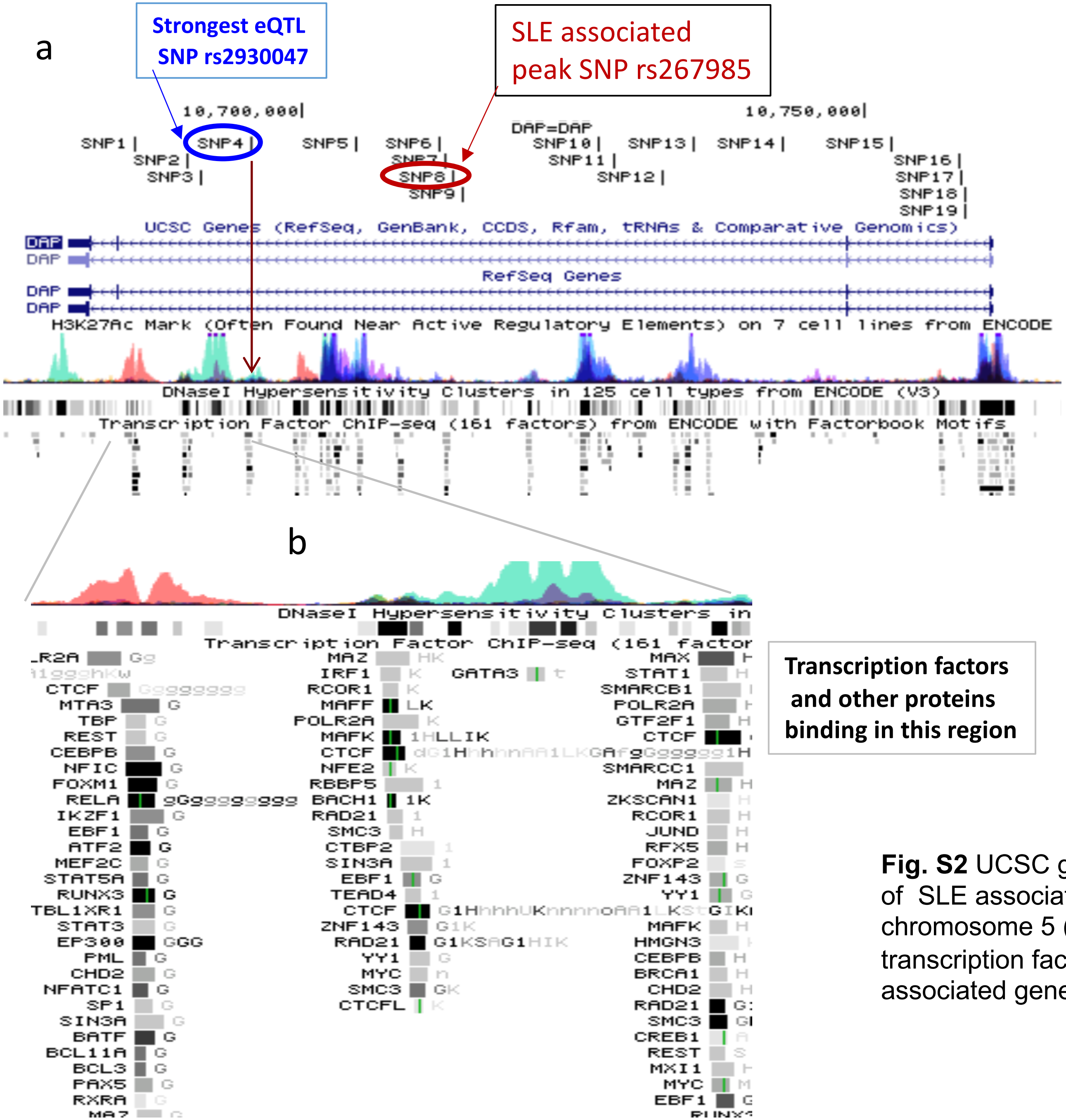

Fig. S2 UCSC genome browser shows distribution of SLE associated 19 DAP1 SNPs on chromosome 5 (a). Zoom in picture in (b) shows transcription factors that may be impacted by SLE associated genetic changes in DAP1 gene.

Fig. S3. DAP1 eQTLwith rs2930047 in multiple data sets

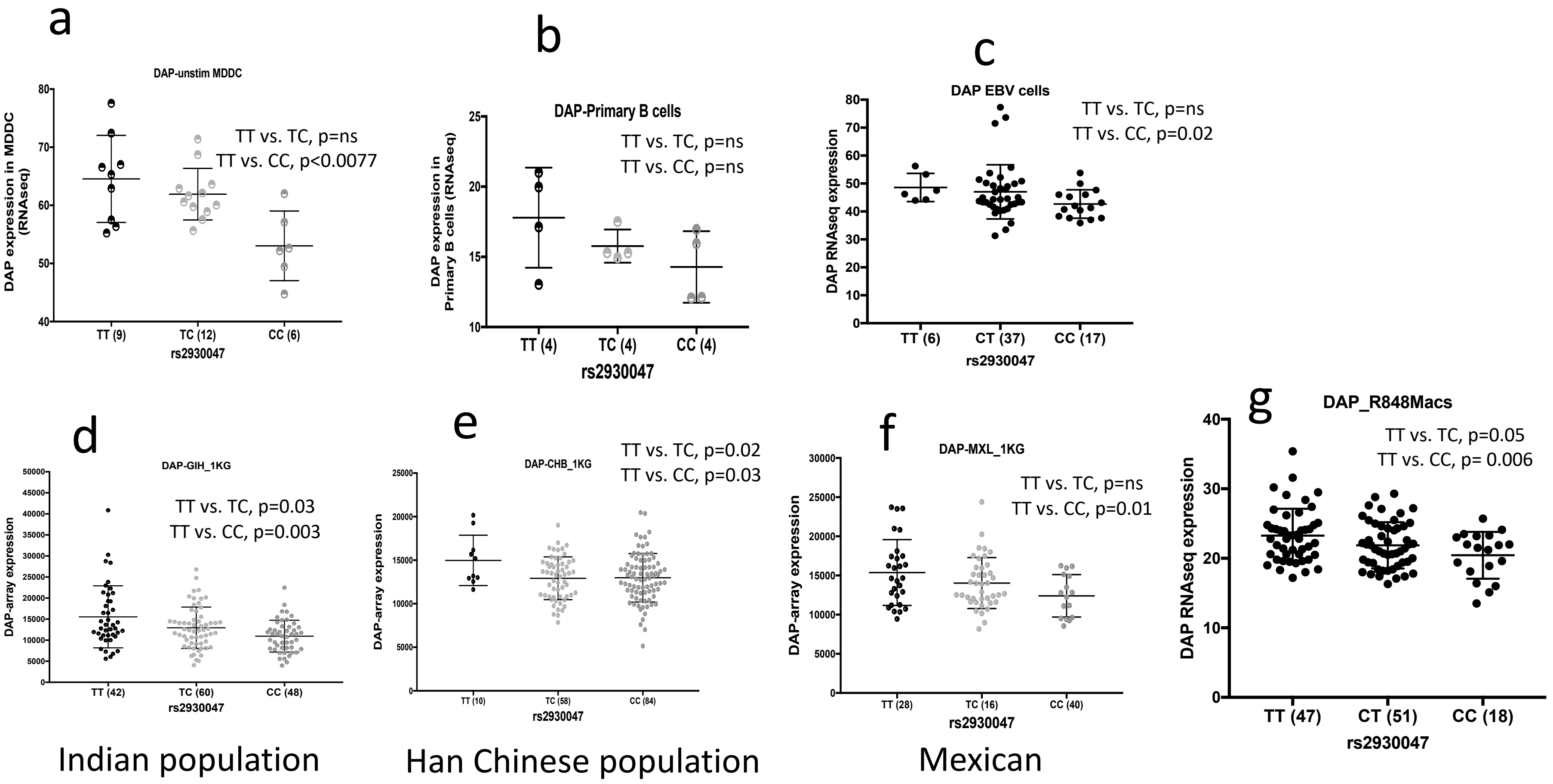

Fig. S3 DAP eQTLwith rs2930047 in unstimulated monocyte derived macrophages (a), in primary B cells from healthy donors (b), in EBV cells (c), in 1000G study participants from Indian ancestry (d), 1000G study participants from Han Chines ancestry (e) 1000G study participants from Mexican ancestry (f) and R848 TLR pathway stimulated macrophages from present study (g)

Fig. S4. DAP 1 reduction and autophagy induction data in PBMCs and monocytes of healthy donors with TT or CC genotype.

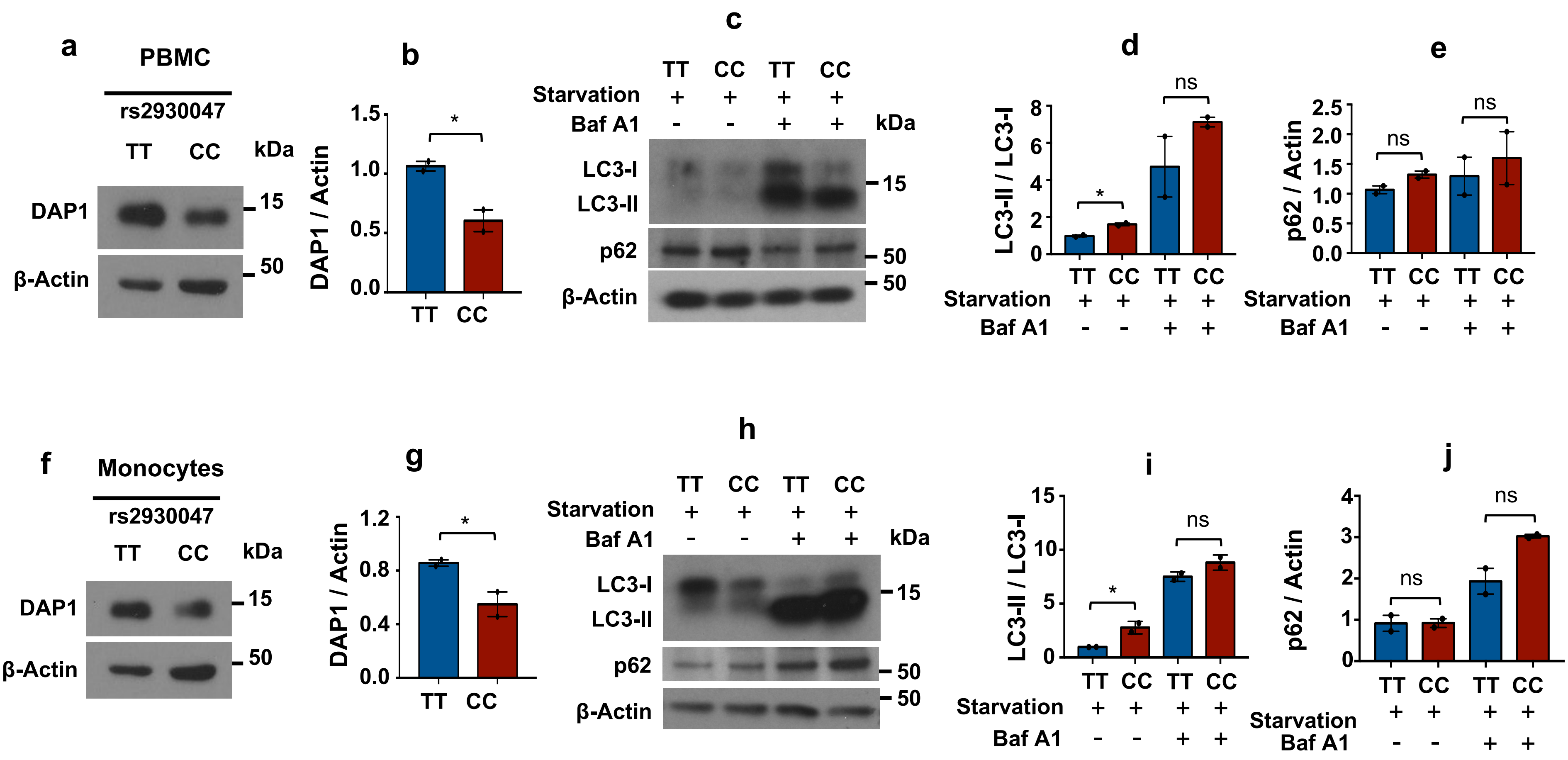

Fig. S4. **Panel (a -e)** PBMCs from healthy donor with rs2930047 protective genotype (TT) or donor with rs2930047 risk genotype (CC) incubated with EBSS in the absence or presence of 100 nM Bafilomycin A1 (Baf A1) for 4 hours. Cell lysates were analyzed by western blot using anti-DAP1, anti-p62 and anti-LC3 antibodies. β-Actin was used as a loading control. Panel **(f and j)** Monocytes from healthy donor with protective genotype (TT) or donor with risk genotype (CC) incubated with M-CSF (50ng/mL) in the absence or presence of 100 nM Baf A1 for 14 hours. Cell lysates were analyzed by western blot using indicated antibodies. Bar graph represents the average ± SEM of two independent experiments. Error bars: SEM; *, $P < 0.05$. ns, not significant. Student's *t* test.

Fig. S5. Gene expression status of some key genes in immune system functions

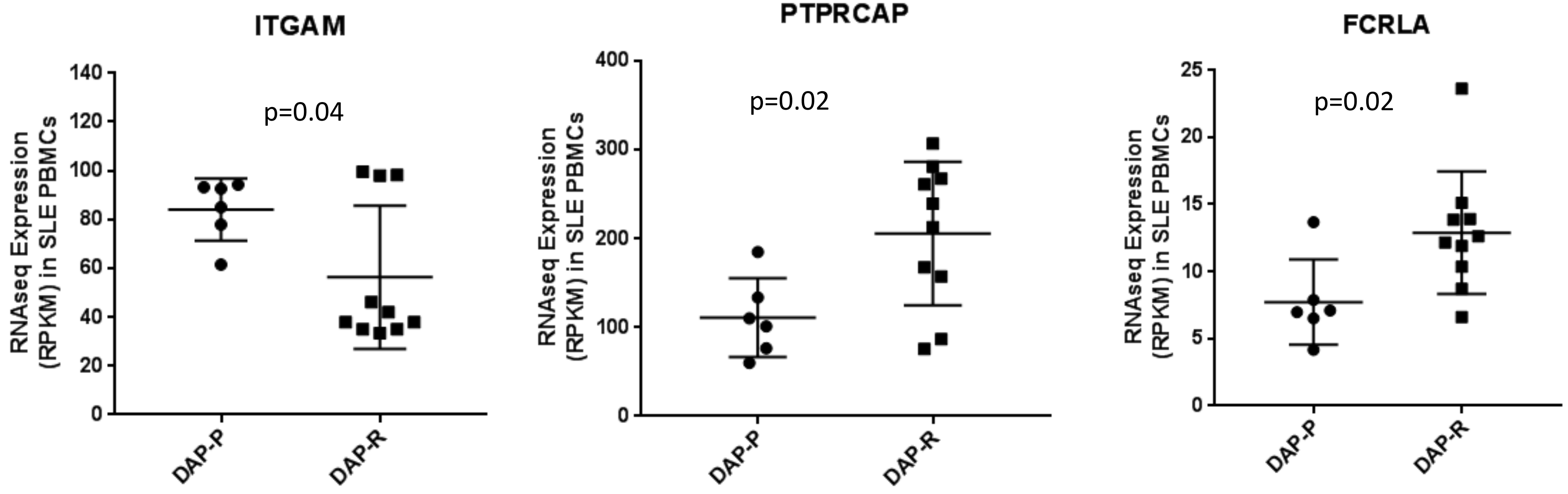

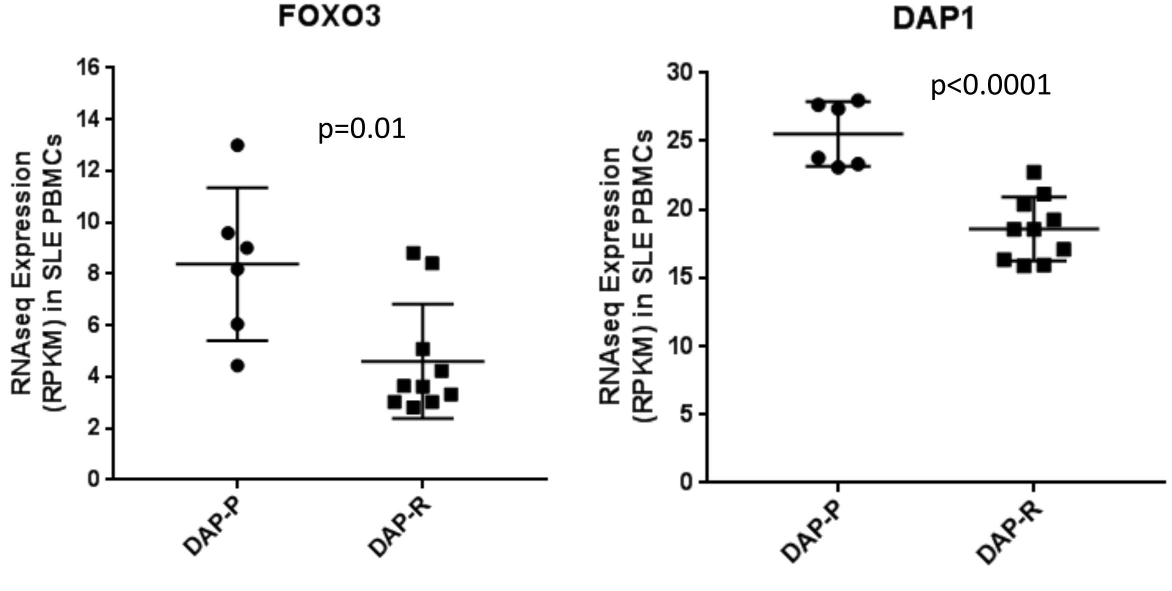

Fig. S5. Shows expression level of previously known SLE risk genes i.e. *ITGAM, FOXO3*, some key molecules that play role in B and T cell activation i.e. *FCRLA* and genes of interest for present study i.e. DAP1 in DAP-P (protection) and DAP-R (Risk) genotype.

Fig. S6. Enrichment of *sm* and *snRNP* targeted antibodies in DAP1 SLE risk allele

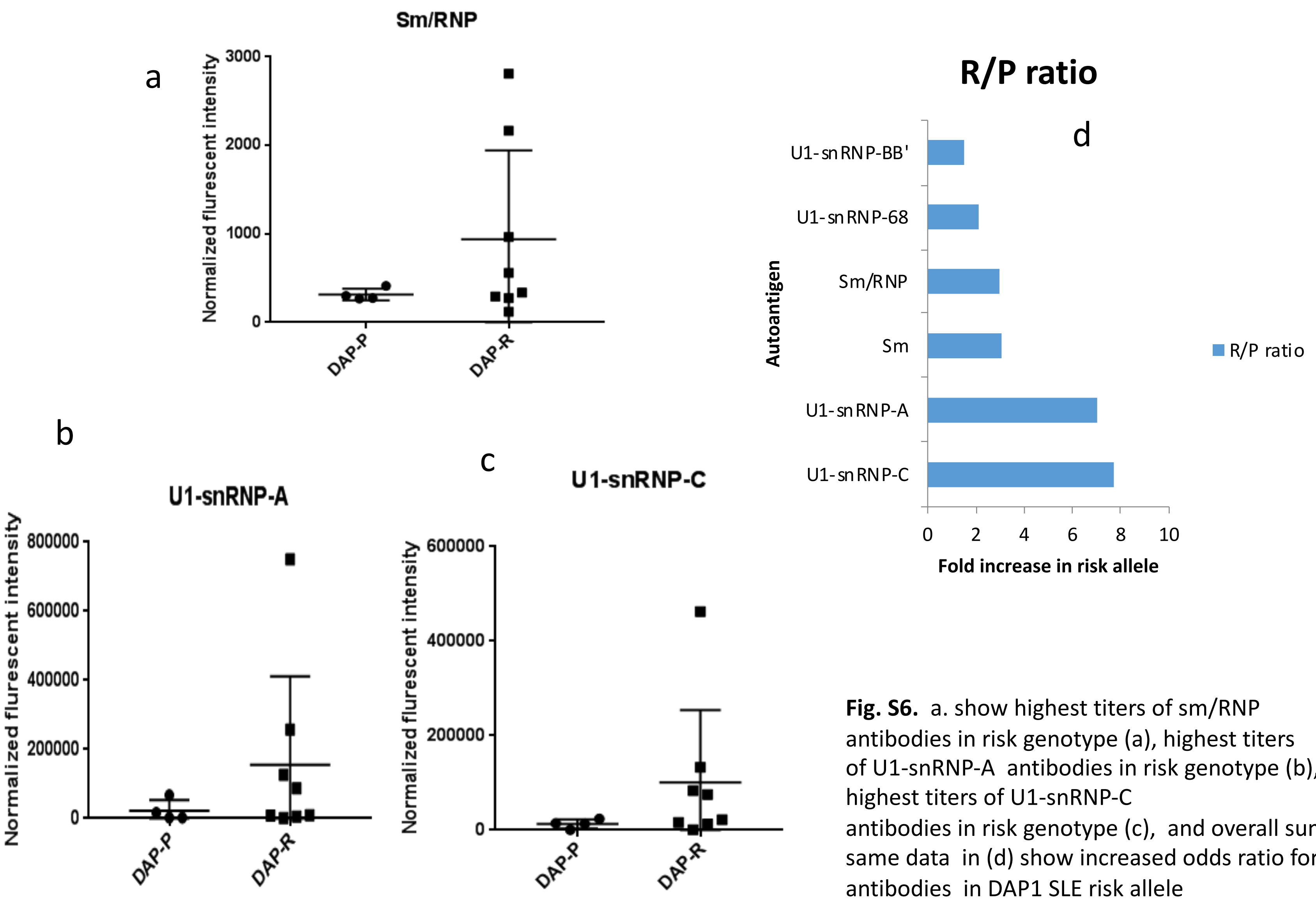

Fig. S6. a. show highest titers of sm/RNP antibodies in risk genotype (a), highest titers of U1-snRNP-A antibodies in risk genotype (b), highest titers of U1-snRNP-C antibodies in risk genotype (c), and overall summary of same data in (d) show increased odds ratio for these antibodies in DAP1 SLE risk allele

Fig. S7. Association of non-nuclear autoantigen signatures with DAP1 SLE risk allele

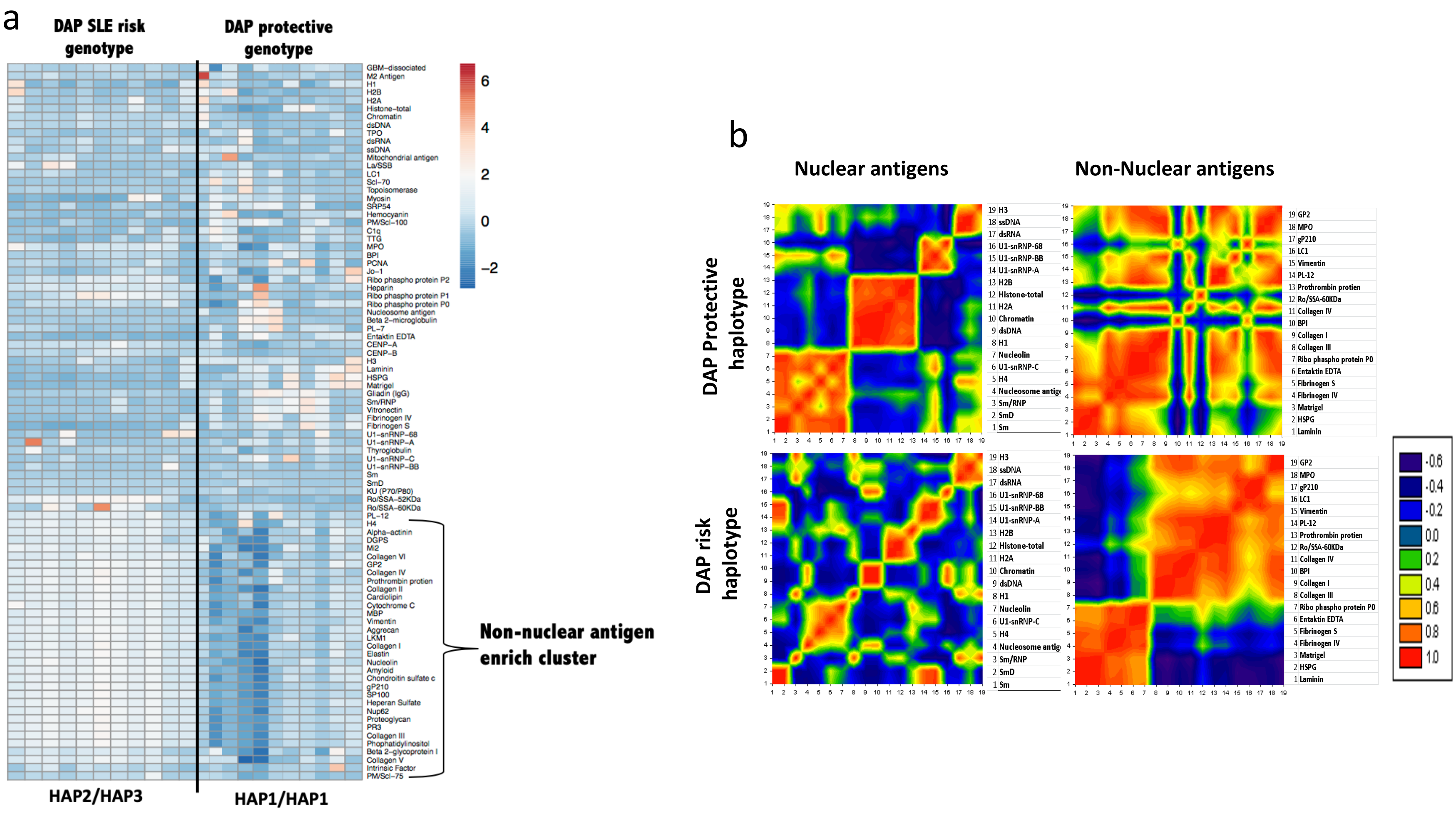

Fig. S7. Autoantibodies in patients with and without DAP1 risk haplotype

Data on SLE patients with and without DAP1 risk haplotype. a. Heatmap show individuals antigens enriched in DAP1 risk haplotype. b. Clusters analysis show of nuclear and non-nuclear autoantigens in DAP1 SLE risk and protective haplotypes, illustrates more stable clusters of non-nuclear antigens in DAP1 risk haplotype. Heatmap color bars indicate the strength of correlation (Pearson r) among autoantigens, with blue color indicating poor and red color indicating strong correlation.

Fig. S8. Upregulated expression of antigen presentation pathway molecules in DAP1 risk genotype

a. LCLs from 1000 Genome study

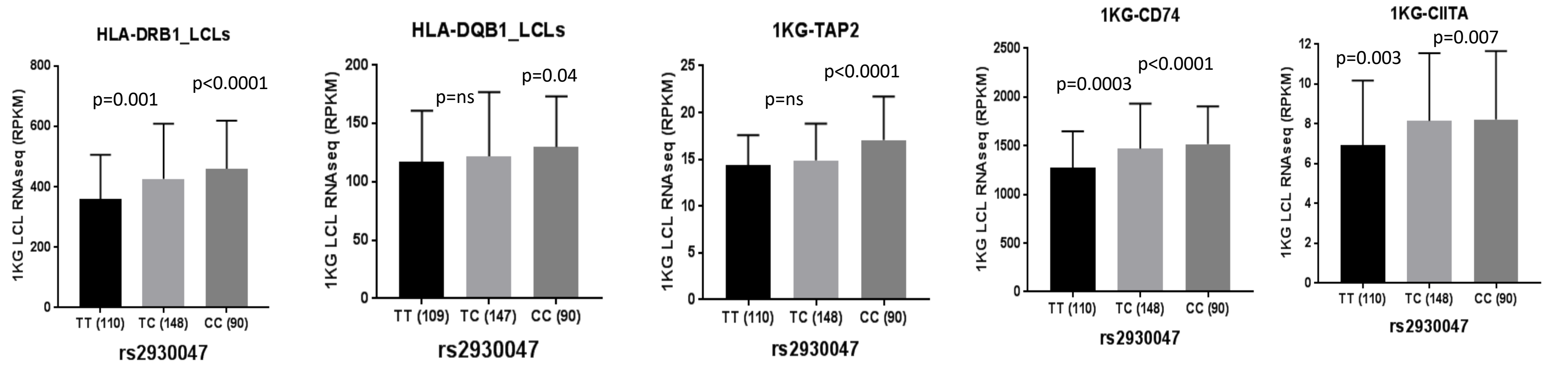

b. Primary B cell from healthy donors-present study

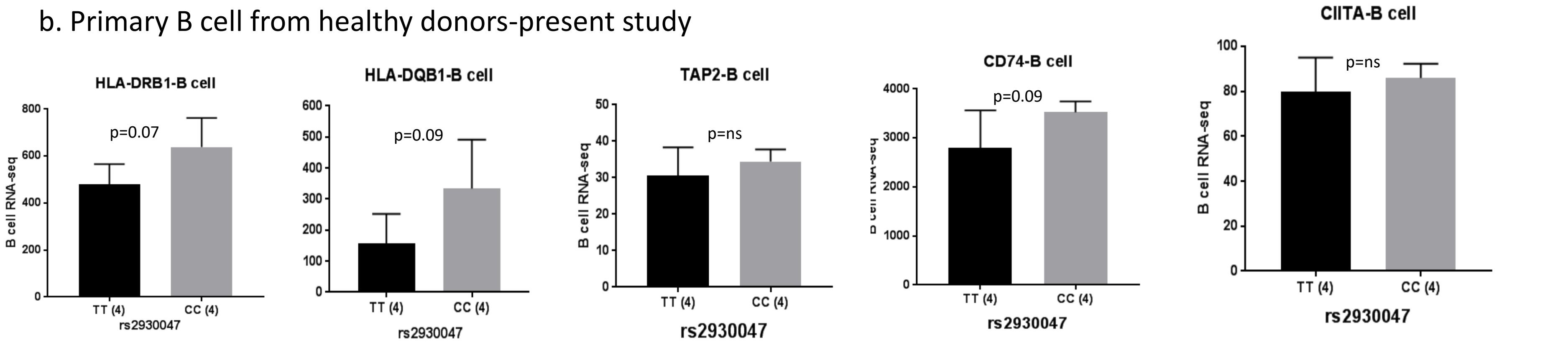

Fig. S8 Show expression of HLA-class II genes and its regulators in LCLs from 1000 genome study (a) and primary B cells of healthy donors with DAP1 risk genotype (b). RNAseq and genotyping data on LCL data is from 1000 Genome study and B cells data were generated in the present study. Note: TT and CC genotype of rs2930047 SNP represent DAP1 protective and risk allele, respectively.

Fig. S9. Transcription levels of DAP1 in primary B cells of healthy donors with protective and risk DAP1 allele.

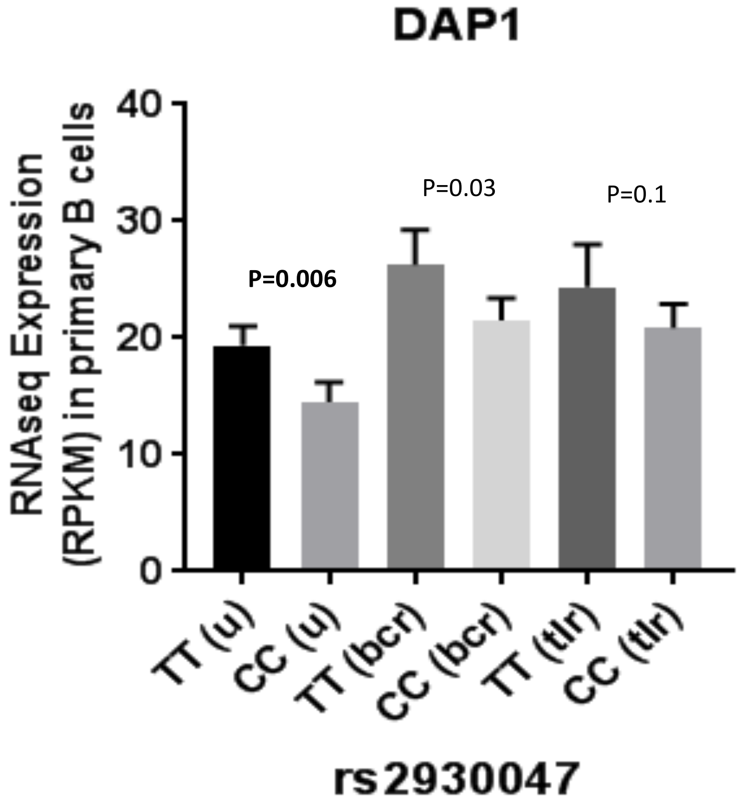

Fig. S9. Expression level of DAP1 are compared for unstimulated (u), B cell receptor stimulated (bcr) and TLR stimulated (tlr) cellular states.

Fig. S10. Transcription levels of SLE associated autophagy genes in PBMCs (RNAseq)

a

Table 1. The list of variants in autophagy-related genes associated with the susceptibility to S

Eur. J. Immunol. 2019. 49: 523–533

Gene	Chr	SNP	Population	Category	Reference
ATG5	6	rs573775	Caucasians	GWAS	[12]
		rs6568431	Caucasians, Chinese	GWAS	[12]
		rs2245214	Caucasians	Candidate gene	[13]
		rs548234	Chinese	GWAS	[14]
		rs6937876	Chinese	Candidate gene	[15]
ATG16L2	11	rs11235604	Koreans	GWAS	[16]
DRAM1	12	rs4622329	Chinese	GWAS	[17]
CDKN1B	12	rs34330	Chinese	GWAS	[17]
CLEC16A	16	rs12708716	Caucasians	GWAS	[13]
		rs73885319		GWAS	
APOL1	22	rs60910145	African American	Candidate gene	[18]
		rs71785313		Candidate gene	
MTMR3	22	rs9983	Chinese	Candidate gene	[22]
ATG16L1	2	rs2241880	Chinese	Candidate gene	[15]
ATG7	3	rs11706903	Chinese	Candidate gene	[15]
IRGM	5	rs13361189	Chinese	Candidate gene	[15]
		rs10065172	Chinese	Candidate gene	[15]
LRRK2	12	rs2638272	Chinese	Candidate gene	[23]
MAP1LC3B	16	rs933717	Chinese	Candidate gene	[24]

Fig. S10. a. Show the published list of known SLE-autophagy genes from literature. b. Transcription levels some of these genes in heathy and SLE PBMCs RNAseq data. Note: Top panel of healthy and SLE were sequenced on a separate RNAseq run than bottom panel, so, scales of RPKM values are quantitatively different between two experiments but comparable within each set.

b

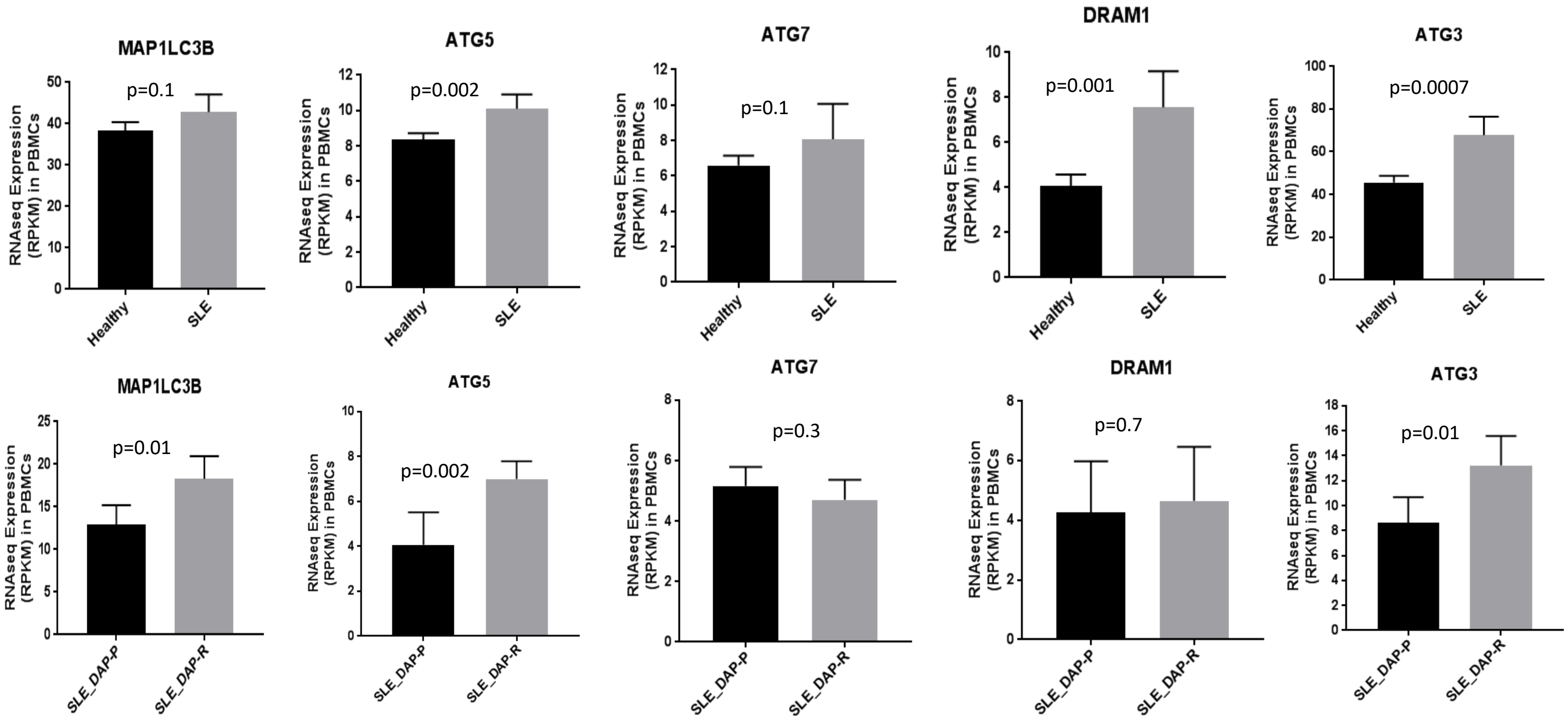

Fig. S11. HAP3 defining variants impact transcription factors that have highest expression in T & B cells

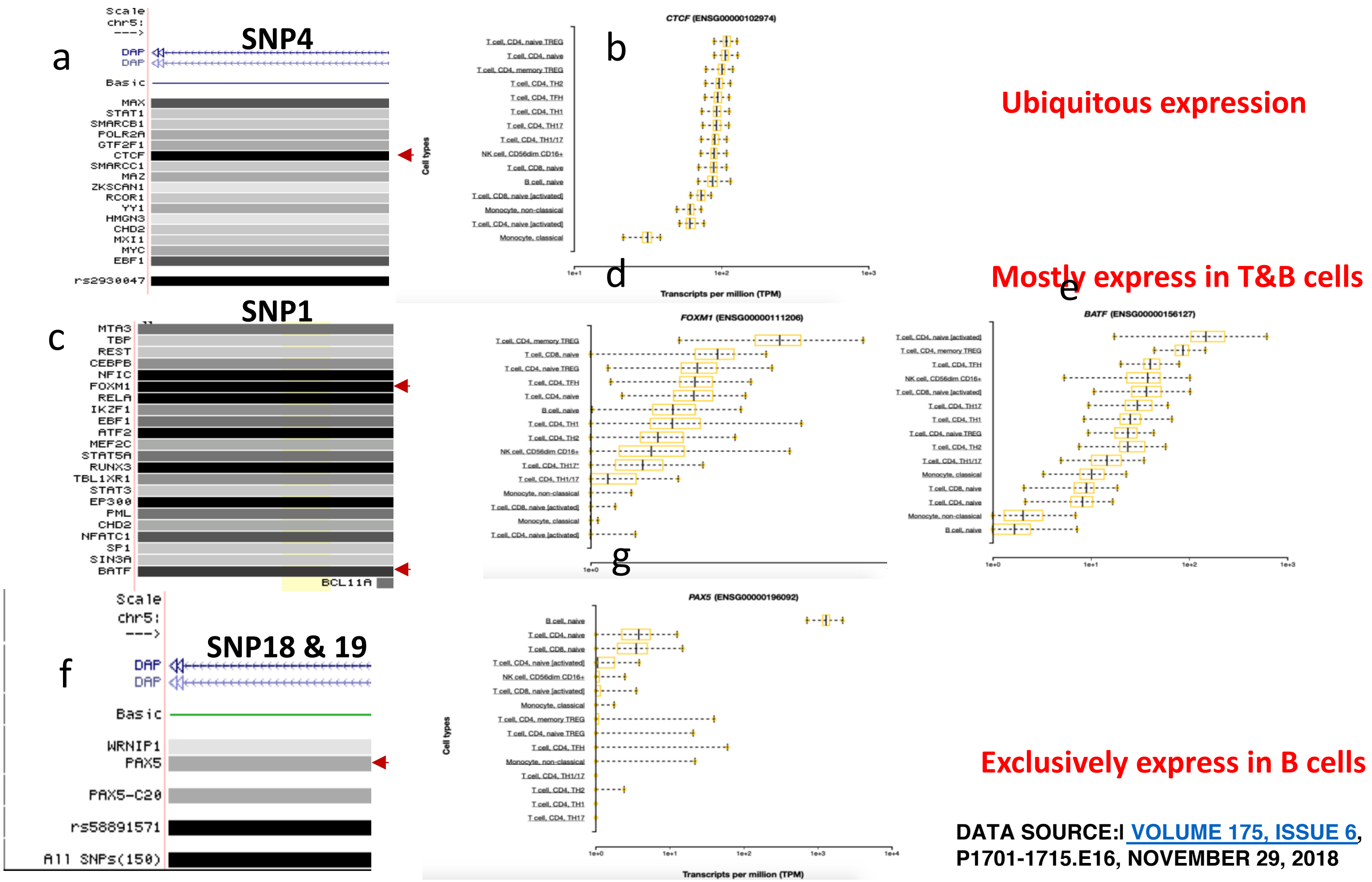

Fig. S11 a. UCSC genome browser snap shot showing position of SNP4 rs2930047 and transcription factors (TFs) i.e. CTCF and other proteins whose binding may be impacted by this variant, b. shows immune cell expression of CTCF, c. UCSC snap shot across SNP1 rs3797111 showing multiple TFs whose binding may be impacted by this variant. D & e. shows higher expression FOXM1 and BATF proteins in T cells, suggesting likely impact of this variant in T cells, f. UCSC snap shot across SNP18 & 19 rs58891571 , rs58583280 Showing interaction of PAX5 protein at this position, panel g. shows that PAX5 exclusively express in B cells, suggesting likely impact of these variants in B cells.

Fig. S12. Statistical power of study

A B

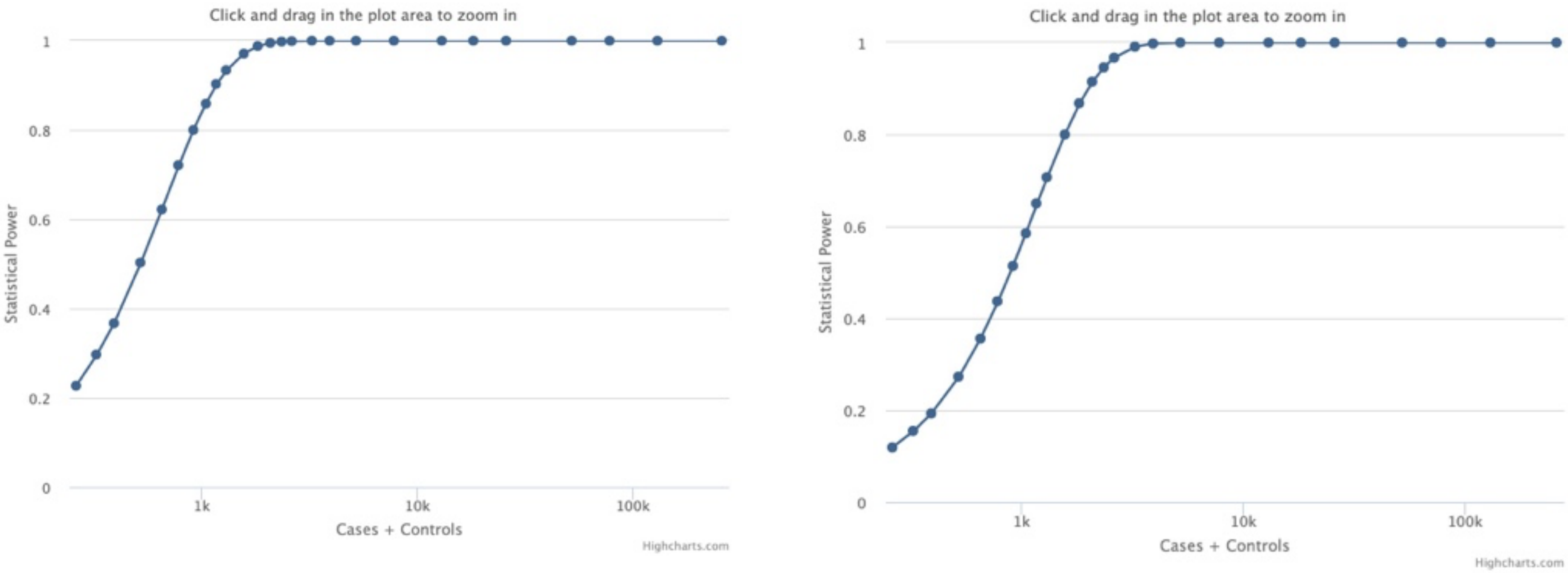

Fig. S12. Statistical power analysis on 1255 Cases and 777 Controls, we obtained a power of 99.4% for SNP analysis (A) and 90% for haplotype analysis (B)

Fig. S13a. Uncropped blots related to main Figure 3.

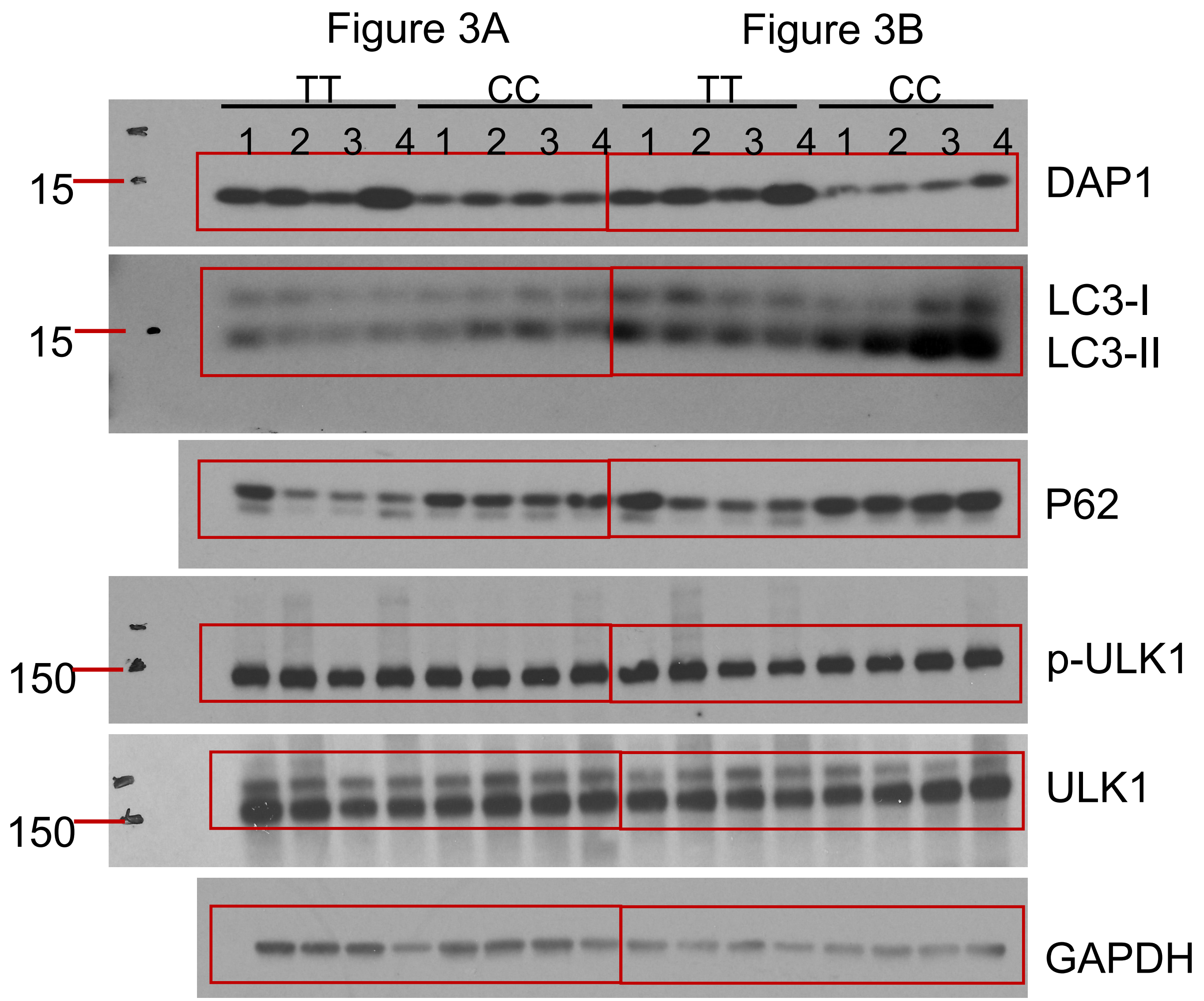

Fig. S13. LCLs from four donors with protective genotype (TT) or four donors with risk genotype (CC) incubated with RPMI in the absence or presence of 100 nM Bafilomycin A1 for 4 hours. Cell lysates were analyzed by western blot using indicated antibodies.

Fig. S13b. Uncropped blots related to main Figure 3.

DAP1

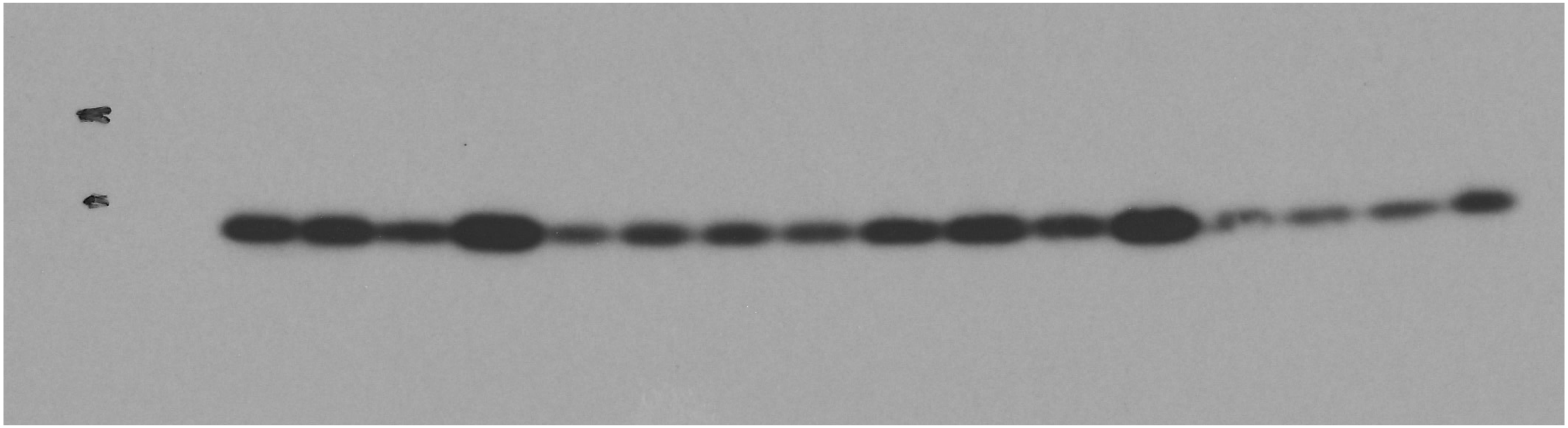

LC3

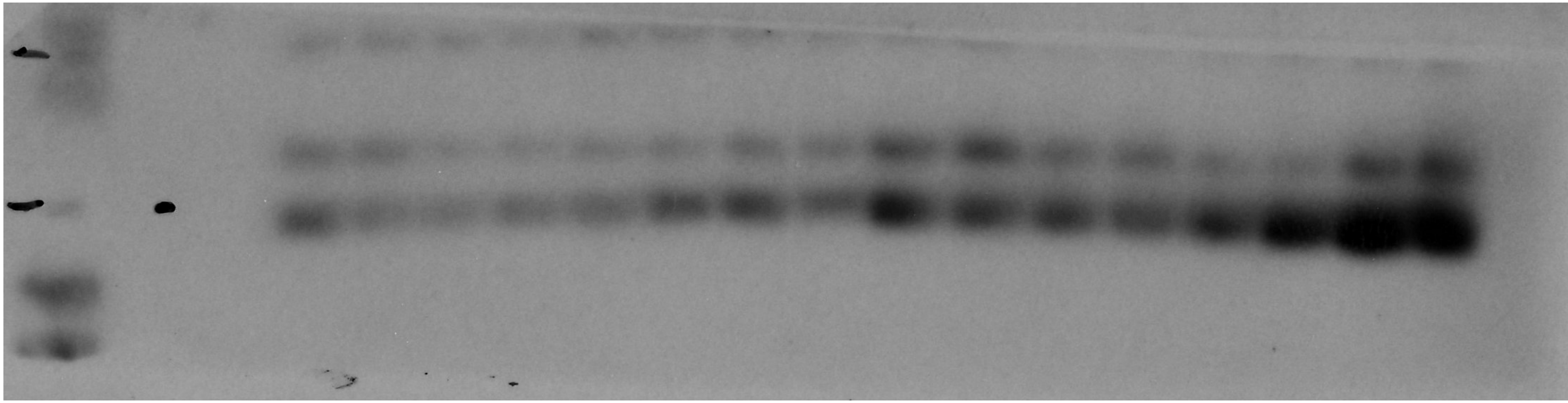

Fig. S13c. Uncropped blots related to main Figure 3.

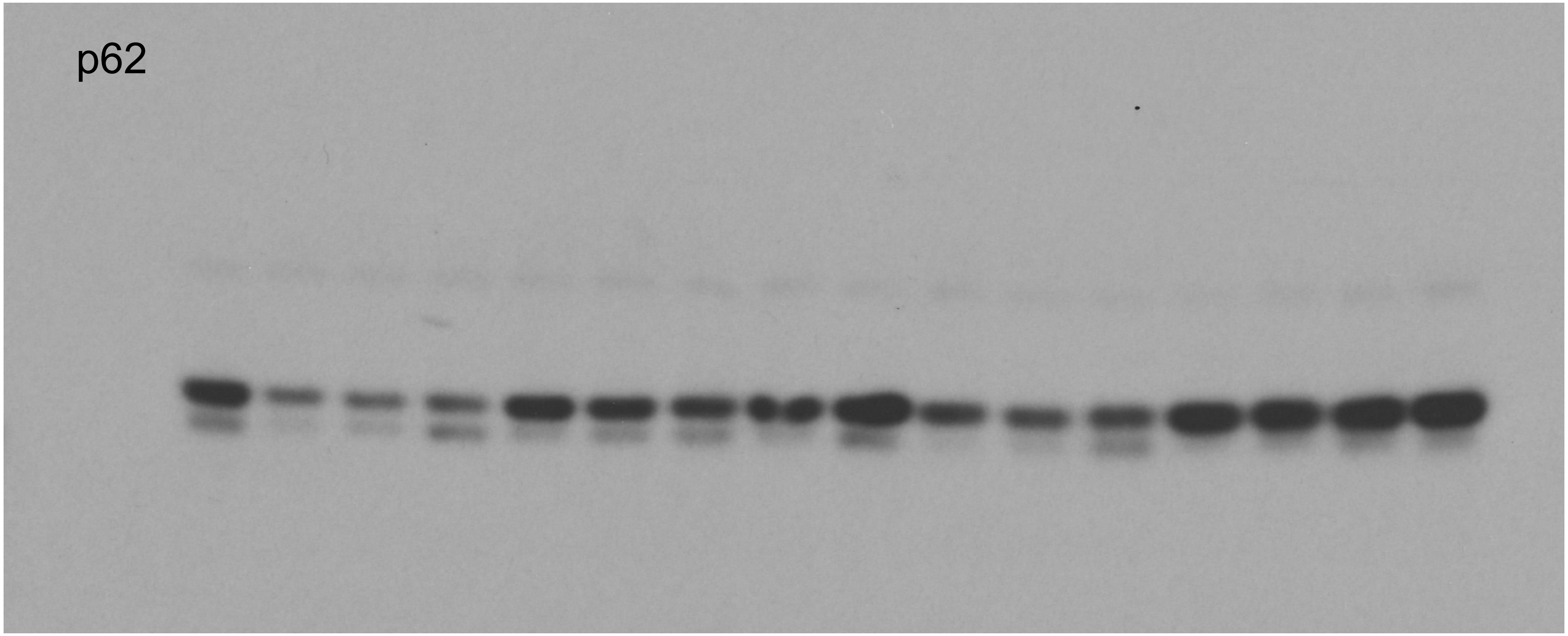

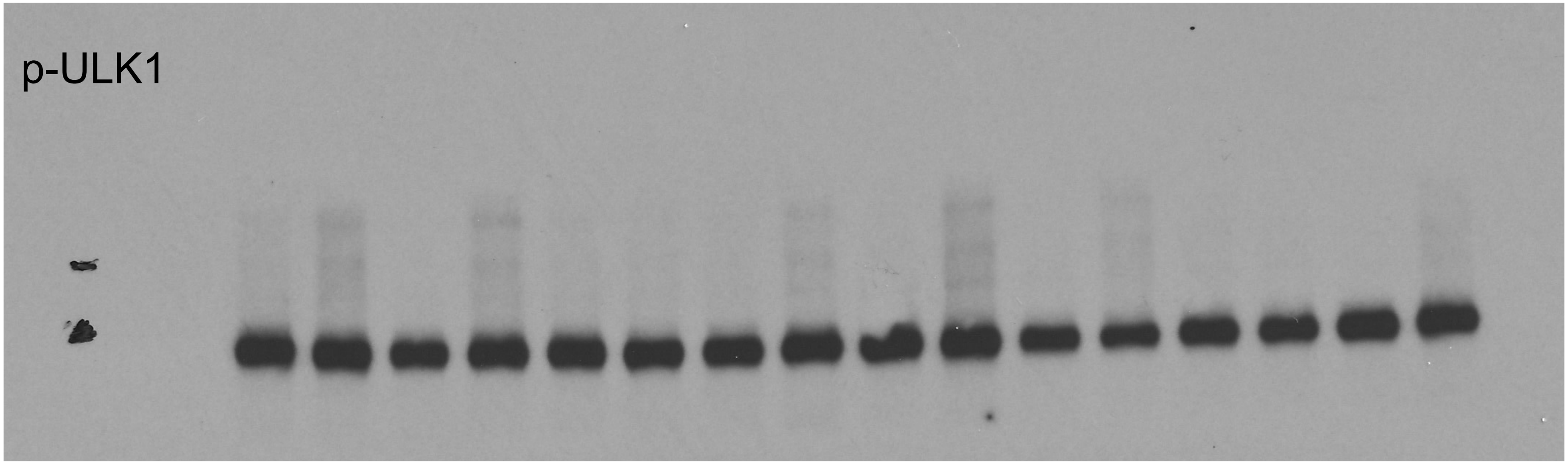

Fig. S13d. Uncropped blots related to main Figure 3.

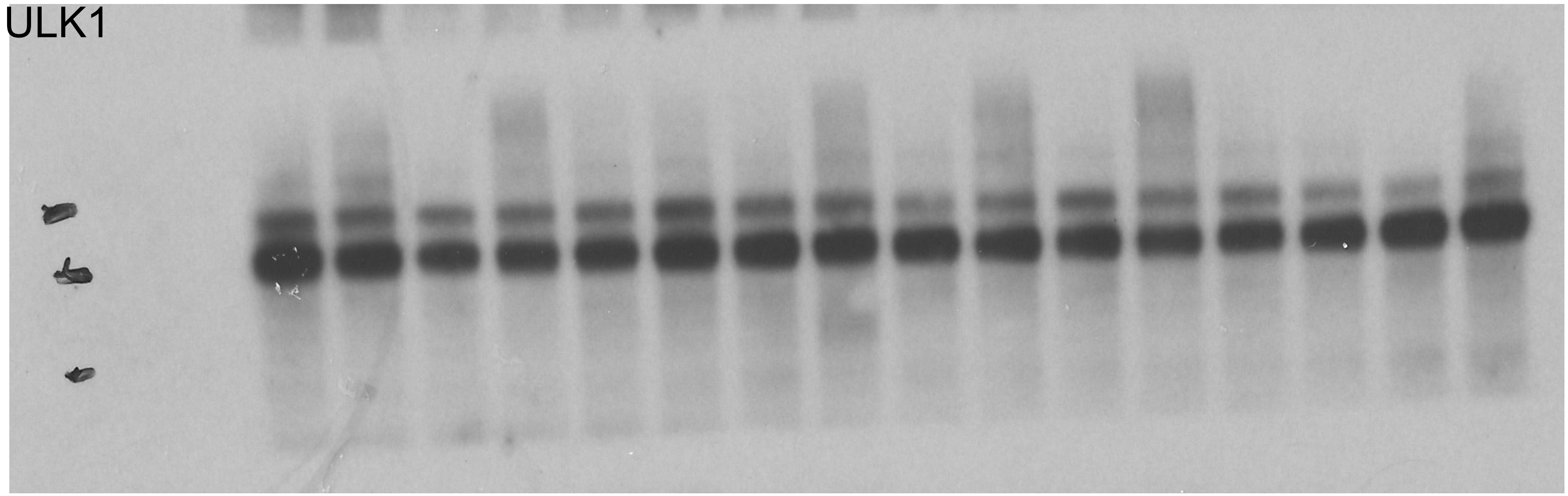

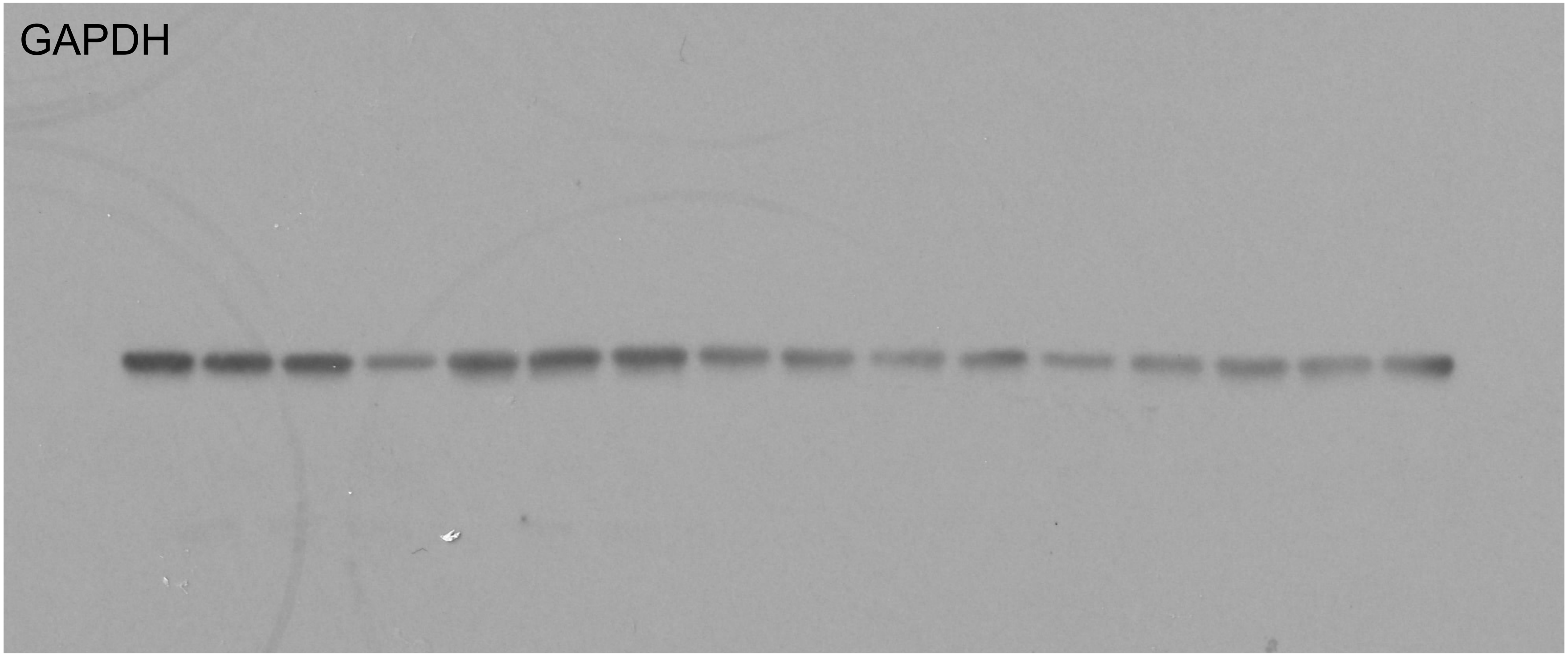

Supplement: Supplementary file 2 — Additional file 2: Fig. S1. DAP1 genetic association with SLE. Fig. S2. Genomic position of SLE associated DAP1 variants. Fig. S3. DAP1 eQTL with rs2930047. Fig. S4. Autophagy induction data in PBMCs and monocytes. Fig. S5. Gene expression data. Fig. S6 Sm and snRNP antibodies in DAP1 SLE risk allele. Fig. S7. Heatmap of non-nuclear autoantigen signatures. Fig. S8 Expression of antigen presentation pathway. Fig. S9 DAP1 expression in primary B cells. Fig. S10 RNA-Seq data in PBMCs. Fig. S11. HAP3 defining key variants. Fig. S12. Statistical power analysis. Fig. S13. Uncropped blots. [file 13059_2020_2184_MOESM2_ESM.pdf]
